# Concurrent use of opioids and stimulants and risk of fatal overdose: A cohort study

**DOI:** 10.1186/s12889-022-14506-w

**Published:** 2022-11-15

**Authors:** Heather Palis, Chloé Xavier, Sabina Dobrer, Roshni Desai, Kali-olt Sedgemore, Marnie Scow, Kurt Lock, Wenqi Gan, Amanda Slaunwhite

**Affiliations:** 1 BC Centre for Disease Control, 655 W 12th Ave, Vancouver, BC V5Z 4R4 Canada; 2 University of British Columbia, Department of Psychiatry, 2255 Wesbrook Mall, Vancouver, BC V6T 2A1 Canada; 3 Coalition of Peers Dismantling the Drug War, Vancouver, Canada; 4 University of British Columbia, School of Population and Public Health, 2206 E Mall, Vancouver, BC V6T 1Z3 Canada

**Keywords:** Opioid use, Stimulant use, Concurrent substance use, Overdose, Fatal overdose, Cohort study

## Abstract

**Background:**

Stimulant use has been rising among people with opioid use disorder in recent years in North America, alongside a parallel rise in illicit drug toxicity (overdose) deaths. This study aimed to examine the association between stimulant use and overdose mortality.

**Methods:**

Data from a universal health insurance client roster were used to identify a 20% random general population sample (aged ≥12) in British Columbia, Canada between January 1 2015 and December 31 2018 (*N* = 1,089,682). Provincial health records were used to identify people who used opioids and/or stimulants. Fatal overdose observed during follow-up (January 12,015- December 312,018) was retrieved from Vital Statistics Death Registry and BC Coroners Service Data. Potential confounders including age, sex, health region, comorbidities and prescribed medications were retrieved from the provincial client roster and health records.

**Results:**

We identified 7460 people who used stimulants and or opioids. During follow-up there were 272 fatal overdose events. People who used both opioids and stimulants had more than twice the hazard of fatal overdose (HR: 2.02, 95% CI: 1.47-2.78, *p* < 0.001) compared to people who used opioids only. The hazard of death increased over time among people who used both opioids and stimulants.

**Conclusions:**

There is an urgent need to prioritize the service needs of people who use stimulants to reduce overdose mortality in British Columbia. Findings have relevance more broadly in other North American settings, where similar trends in opioid and stimulant polysubstance use have been observed.

**Supplementary Information:**

The online version contains supplementary material available at 10.1186/s12889-022-14506-w.

## Background

Illicit drug toxicity (i.e. overdose) death has been on the rise in recent years in North America. In Canada there were 26,690 opioid toxicity deaths between Jan 2016 and Sep 2021, and more than half (58%) of these deaths in 2021 also involved a stimulant [1]. Furthermore, among all stimulant toxicity deaths in 2021, nearly 90% also involved an opioid. These data reflect a population-level trend toward increasing concurrent use of opioid and stimulants. Though the public health emergency of drug poisoning deaths (overdose crisis) has impacted most provinces and territories in Canada, British Columbia (Canada’s third most populous province) has consistently reported the highest rates of illicit drug toxicity deaths in the country, more than double the national average in 2021 [1].

Provincial death data reveal an increasing prevalence of methamphetamine alongside opioids in toxicity records. For example, methamphetamine was detected in only 14% of deaths in 2012 however between 2019 and 21 it was detected in approximately 42% of deaths [2].While the detection of cocaine in drug toxicity deaths has generally been declining in recent years, it remained involved in 44% of illicit drug toxicity deaths in 2020 [2]. This trend has also been detected among people accessing harm reduction sites across British Columbia (BC), where methamphetamine is the most commonly reported substance used among more than 70% of respondents [3]. This analysis also revealed that people who reported opioid use had three times the odds of concurrent methamphetamine use compared to people who did not report using opioids [3]. These patterns have also been observed outside BC [4]. For example, a recent national study of people with opioid use disorder (OUD) in the United States revealed a significant rise in reported methamphetamine use from 18.8% in 2011 to 34.2% in 2017 [5]. This rise in stimulant use among people who use opioids in North America has been referred to as “twin epidemics” [5].

While evidence-based treatments for people with OUD are available to reduce risk of illicit drug toxicity events, including opioid agonist treatment (OAT) [6], engagement in this treatment is often limited among people who use stimulants [7]. For example, prior studies have found that people who concurrently use cocaine or methamphetamine alongside opioids have reduced retention rates in OAT compared to people with OUD alone [8]. Given the protective effect of OAT on overdose, where stimulant use interferes with OAT engagement, overdose risk may be elevated [7, 9]. Furthermore, studies have suggested that the concurrent use of opioids and stimulants is common among people who access harm reduction sites in BC [10]. Motivations for co-use have been described in a variety of studies, and have ranged from social influences, to seeking to reduce opioid withdrawal, to imrpvoing functionality, energy, or wakefulness [11–13]. One study has also revealed a dangerous misperception among people who co-use opioids and stimulants, that stimulants can have a protective against opioid overdose [14]. This is untrue, in fact, studies have identified that polysubstance use elevates overdose risk especially where the quality and potency of the substance is unknown [15].

Evidence is emerging to support the safety and effectiveness of pharmacological treatments for stimulant use disorder, however their implementation has been limited, often leaving people who use both opioids and stimulants disconnected from care [16]. This is particularly concerning considering people who use both opioids and stimulants are also known to face an elevated burden of concurrent chronic health conditions [17], necessitating access to care in order to avoid premature morbidity and mortality, including from illicit drug toxicity (overdose).

Given recent trends indicating rising stimulant use at a population level, and in particular, its increasing detection in illicit drug toxicity deaths alongside fentanyl in BC, there is a need to investigate the association of stimulant use with illicit drug toxicity deaths. As such, this study aims to estimate the effect of stimulant use, on its own and with opioid use on risk of fatal overdose in BC.

## Methods

### Study design and population

In this prospective cohort study we used a 20% random sample of the general population of British Columbians retrieved from the 2018 British Columbia Provincial Overdose Cohort (BC-ODC) (*N* = 1,089,682) [18]. The BC-ODC was created as part of BC’s response to the declaration of the overdose public health emergency in 2016 and is refreshed annually. The BC-ODC contains administrative health data linked at the patient level through BC’s Client Roster. Registration in the Client Roster is mandatory for all BC residents (including Canadian citizens, permanent residents, those on visas > 6 months and their dependents) to access provincial universal health insurance. Persons with cancer and palliative diagnoses, and children under the age of 12 years were excluded resulting in a final sample of 752,064. Among the 752,064 people, 7460 (about 1%) were identified as persons who used stimulants, opioids, or both. All data sources contained in the BC-ODC are outlined in detail in the Supplement. This analysis has not been pre-registered and findings should be considered exploratory.

## Study variables

### Exposure

The main exposure of interest was type of substance use, identified using International Classification of Diseases (ICD) 9 codes (primary care visits) and ICD10 codes (hospitalizations) for opioid or stimulant use disorder (See supplement). One primary care or one hospitalization between January 1 2010 and December 312,018 was used to indicate use of stimulants and/or opioids. This is a less stringent algorithm than has been applied in prior studies using data from the BC-ODC [19, 20](one hospitalization or two primary care visits with a relevant ICD9/10 code within 1 year of each other) and was applied with the goal of reducing underestimation of the occurrence of opioid and/or stimulant use. If a person had an opioid or stimulant related primary care or hospitalization record prior to the study period, January 1st 2015 was used as a baseline date. If a person had an opioid or stimulant related health encounter during the study period, the date of this encounter was used as the baseline date.

### Outcome

Fatal overdose events were determined using BC Coroners Service data on open investigations (toxicology pending) and closed illicit drug toxicity deaths, and Vital Statistics deaths registry using ICD9 (primary care visits [21]) or ICD10 (hospitalizations [22]) codes indicating drug poisoning by opioids or related narcotics. The definition also includes a drug related overdose algorithm which identifies deaths from administrative databases which lie between the start and end data of identified overdose episodes. Toxicology data are available for closed cases of death only (See Supplement).

## Baseline characteristics

### Demographics

Persons were described by sex (male or female), age at baseline in years (< 30, 30–39, 40-49, 50+), and Health Authority of residence. Health Authorities (HA) are the organizations primarily responsible for health service delivery in BC. The five regional HAs deliver health services to meet the needs of the population within their respective geographic regions. All demographic variables were derived based on data contained in the provincial BC client roster at baseline (See Supplement).

### Comorbidities

Elixhauser comorbidity index was used to provide a summary of comorbidities and was calculated using 31 categories of disease recorded in hospitalization data (ICD10 codes) during the exposure period. Based on the distribution of the data, this index was divided into 4 groups (none, 1, 2, 3 or more). Various combinations of comorbidity covariates were explored in the modeling and results were consistent (See Supplement).

### Prescribed medications

Prescribed medication history was assessed for opioids prescribed for pain, benzodiazepines, z-drugs, and sedative medications (non-opioids/non-benzodiazepines). The impact of OAT access was also examined only among people eligible to receive this treatment (i.e. people in the opioid only group or in the opioid and stimulant group). Prescription data were derived from PharmaNet [23], the provincial drug dispensation database, within 30 days prior to baseline. PharmaNet is a province-wide network that links all BC pharmacies to a central data system. Every prescription dispensed in community pharmacies is entered into this system. Medications included in each category are listed in the Supplement.

## Data analysis

### Descriptive analysis methods

Pearson’s chi-squared test (χ2) for categorical data was used for the comparison of baseline characteristics by substance use type (stimulant use, opioid use, both), and illicit drug toxicity death.

### Time to event analysis

Kaplan-Meier curves using the log-rank test was used to estimate and compare survival between substance use types (stimulant use, opioid use, both).

### Cox proportional hazards models

Prior studies have demonstrated that factors such as sex, age, comorbidities, substance use history, and concurrent prescriptions can contribute to risk of overdose and overdose mortality [24–26]. As such, a series of confounding Cox proportional hazard models were performed to evaluate the relationship between type of substance use and illicit drug toxicity death. The proportionality assumption for the Cox models was evaluated using Kaplan–Meier survival curves for all baseline characteristics. This approach suggested that the proportional hazard assumption held for all characteristics. The log(−log(survival)) versus log of survival time for categorical variables and variables with time interactions were tested. No issues with the proportionality assumption were identified. Participants who had missing data on any variables of interest were excluded from the final model. Characteristics of included vs. excluded participants are presented in the supplement. In order to assess the overtime changes in fatal overdose risk a series of proportional cox models for each year of study (2015-2018) were assessed. For each year, people who survived to the end of the prior year were included. Analyses were repeated by sex and by age group, as well as with and without the inclusion of the OAT variable (See Supplement).

## Results

The sample included 7460 people including people in the stimulants only (38.0%), opioids only (38.9%), or both (23.1%) groups between January 12,010 and December 312,018. There were significant differences in demographic, comorbidity, and medication access characteristics by substance use type. There were significantly more males among people who used stimulants (64.2%) compared to opioids (58.6%) and to people who used both (60.5%). There was a relatively consistent age distribution across substance use types, while people in the stimulant use only group were overrepresented among the < 30 age group and people in the opioid use only group were overrepresented among the 50+ age group. People who used both opioids and stimulants were more likely to have 3 or more comorbidities. People who used opioids only were more likely to receive benzodiazepines, z drugs, sedatives, and pain medications, as compared to people who used stimulants only, and people who use both opioids and stimulants (Table 1).

**Table 1:** Demographic, comorbidity, prescription drug use characteristics of sample, by substance use type.

	Stimulant use only N(%)	Opioid use only N(%)	Both N(%)	Total N(%)	***P*** value
	2837 (38·0)	2901(38·9)	1722 (23·1)	7460	
**Demographics**					
**Sex**					
Female	1017(35·8)	1202(41·4)	681(39·5)	2900(38·9)	< 0·001
Male	1820(64·2)	1699(58·6)	1041(60·5)	4560(61·1)	
**Age**					
< 30	866(30·5)	593(20·4)	477(27·7)	1936(26·0)	< 0·001
30-39	780(27·5)	716(24·7)	514(29·8)	2010(26·9)	
40-49	610(21·5)	586(20·2)	392(22·8)	1588(21·3)	
50+	581(20·5)	1006(34·7)	339(19·7)	1926(25·8)	
**Health authority of residence**					
Vancouver Costal	733(25·8)	645(22·2)	691(40·1)	2069(27·7)	< 0·001
Vancouver Island	383(13·5)	598(20·6)	153(8·9)	1134(15·2)	
Fraser	886(31·2)	892(30·7)	539(31·9)	2317(31·1)	
Interior	517(18·2)	468(16·1)	239(13·9)	1224(16·4)	
Northern	285(10·0)	156(5·4)	90(5·2)	531(7·1)	
Unknown	33(1·2)	142(4·9)	10(0·6)	185(2.5)	
**Comorbidities**					
**Elixhauser index**					
0	1004(35·4)	1630(56·2)	531(30·8)	3165(42·4)	< 0·001
1	544(19·2)	509(17·5)	359(20·8)	1412(18·9)	
2	635(22·4)	296(10·2)	362(21·0)	1293(17·3)	
3+	654(23·1)	466(16·1)	470(27·3)	1590(21·3)	
**Prescribed medications (Prior 30 days at baseline)**					
Benzodiazepines	305(10·8)	447(15·4)	243(14·1)	995(13·3)	< 0·001
Z drugs	139(4·9)	209(7·2)	110(6·4)	458(6·1)	0·001
Sedatives	1036(36·5)	2150(74·1)	1079(62·7)	4265(57·2)	< 0·001
Opioids for pain	256(9·0)	797(27·5)	269(15·6)	1322(17·7)	< 0·001

During follow-up, 272 people died of overdose (illicit drug toxicity death); 40% were in the opioid and stimulant group, 32% were in stimulants only group, and 27% were in the opioids only group. Approximately 70% of overall drug toxicity deaths were among males while 75% of the deaths in the stimulant use only group were in males. Among people who died, the presence of comorbidities was highest in people in the opioid and stimulant group. A higher proportion of people in the opioid only group had dispensations for sedatives and benzodiazepines (Table 2).

**Table 2:** Demographic, comorbidity, prescription drug use characteristics of people who died of overdose during follow-up, by substance use type.

	Stimulant use only N(%)	Opioid use only N(%)	Both N(%)	Total N(%)	***P*** value
	87 (32·0)	74 (27·2)	111 (40·8)	272(100·0)	
**Demographics**					
**Sex**					
Female	22(25·3)	21(28·4)	32(28·8)	75(27·6)	0·340
Male	65(74·7)	53(71·6)	79(71·2)	197(72·4)	
**Age**					
< 30	21(24·1)	24(32·4)	25(22·5)	70(25·7)	0·170
30-39	14(16·1)	13(17·6)	29(26·1)	56(20·6)	
40-49	28(32·2)	13(17·6)	30(27·0)	71(26·1)	
50+	24(27·6)	24(32·4)	27(24·3)	75(27·6)	
**Health Authority Region**					
Fraser	26(30·0)	25(33·8)	21(18·9)	72(26·5)	0·007
Interior	17(19·5)	13(17·6)	18(16·2)	48(17·6)	
Northern	5(5·6)	–	5(4·5)	14(5·1)	
Vancouver Coastal	25(28·7)	16(21·6)	56(50·5)	97(35·7)	
Vancouver Island	14(16·1)	16(21·6)	11(9·9)	41(15·1)	
**Comorbidities**					
**Elixhauser index**					
0	19(21·8)	34(45·9)	28(25·2)	81(29·8)	0·013
1	21(24·1)	15(20·3)	20(18·0)	56(20·6)	
2	19(21·8)	7(9·5)	22(19·8)	48(17·6)	
3+	28(32·3)	18(24·3)	41(36·9)	87(32·0)	
**Prescribed medications (Prior 30 days at baseline)**					
Benzodiazepines	15(17·2)	15(20·3)	19(17.1)	49(18·0)	0·839
Z drugs	8(9·2)	5(6·8)	12(10.8)	25(9·2)	0·646
Sedatives	48(55·2)	52(70·3)	71(64.0)	171(62·9)	0·135
Opioid for pain	18(20·7)	16(21·6)	24(21.6)	58(21·3)	0·985

**Footnote:** *HR* = Hazard Ratio; No one in the unknown HA reflected in Table 1 had a fatal overdose, as such this group is not reflected in Table 2. The N in Northern Health for opioid use only is suppressed in accordance with Data Sharing Policies due to small N (< 5)

Data were missing for variables of interest among 2.5% (*N* = 185) of all participants (See supplement). The final cox proportional hazards model was run on the sample with no missing data, and revealed that people in the opioid and stimulant group had more than twice the hazard of fatal overdose (HR: 2.02, 95% CI: 1.47-2.78, *p* < 0.001) compared to people in the opioid only group, while there was no significant difference in the hazard for people in the stimulant only group compared to the opioid only group (HR: 1.05, 95%CI: 0.75-1.48, *p* = 0.7644). Females had approximately half the hazard of fatal overdose compared to males (HR: 0.53, 95%CI: 0.40-0.69, *p* < 0.001). After adjusting for all other variables, there were no significant differences in the hazard of fatal overdose by age, health authority, comorbidity index, or prescribed medications (Table 3).

**Table 3:** Unadjusted and adjusted hazard ratio estimates of overdose death by substance use type (Cox proportional hazards model) (*N* = 7275).

	Unadjusted Estimates		Adjusted Estimate	
	HR (95%CI)	P-value	HR (95%CI)	P-value
Substance use type				
Both	1·94 (1·45-2·61)	< 0·001	2·02 (1·47-2·78)	< 0·001
Stimulant	0·99 (0·72-1·35)	0·9322	1·05(0·75-1·48)	0·7644
Opioid	Reference			
Sex-Female	0·54 (0·41-0·70)	< 0·001	0·53 (0·40-0·69)	< 0·001
Age				
< 30	0·92 (0·67-1·28)	0·6249	1·07 (0·75-1·52)	0·7112
30-39	0·68(0·48-0·96)	0·0305	0·74(0·51-1·06)	0·0974
40-49	1·07(0·77-1·48)	0·6988	1·08(0·78-1·51)	0·6335
50+	Reference			
Health Authority				
Fraser	0·68 (0·50-0·92)	0·0117	0·79 (0·58-1·07)	0·1270
Interior	0·85(0·60-1·20)	0·3479	1·01(0·71-1·44)	0·9440
Northern	0·56(0·32-0·99)	0·0449	0·70 (0·40-1·23)	0·2163
Vancouver Island	0·79(0·55-1·14)	0·2131	1·01 (0·69-1·47)	0·9642
Vancouver Coastal	Reference			
Elixhauser index				
None	0·82 (0·61-1·12)	0·2167	0·96 (0·69-1·33)	0·8128
1	0·78(0·56-1·10)	0·1536	0·89(0·63-1·26)	0·5260
2	0·67(0·47-0·95)	0·0248	0·71(0·49-1·01)	0·0564
3+	Reference			
Benzodiazepine	1·27 (0·93-1·73)	0·1317	1·19 (0·85-1·67)	0·3135
Z-drugs	1·35(0·90-2·04)	0·1521	1·21(0·79-1·86)	0·3863
Sedatives	1·23(0·97-1·58)	0·0927	1·04(0·77-1·41)	0·7836
Opioids for pain	1·16(0·87-1·55)	0·3164	1·15(0·82-1·60)	0·4152

Footnote: *HR* = Hazard Ratio; No one in the unknown HA reflected in Table 1 had a fatal overdose, as such this group is not reflected in Table 2

**Fig. 1:**
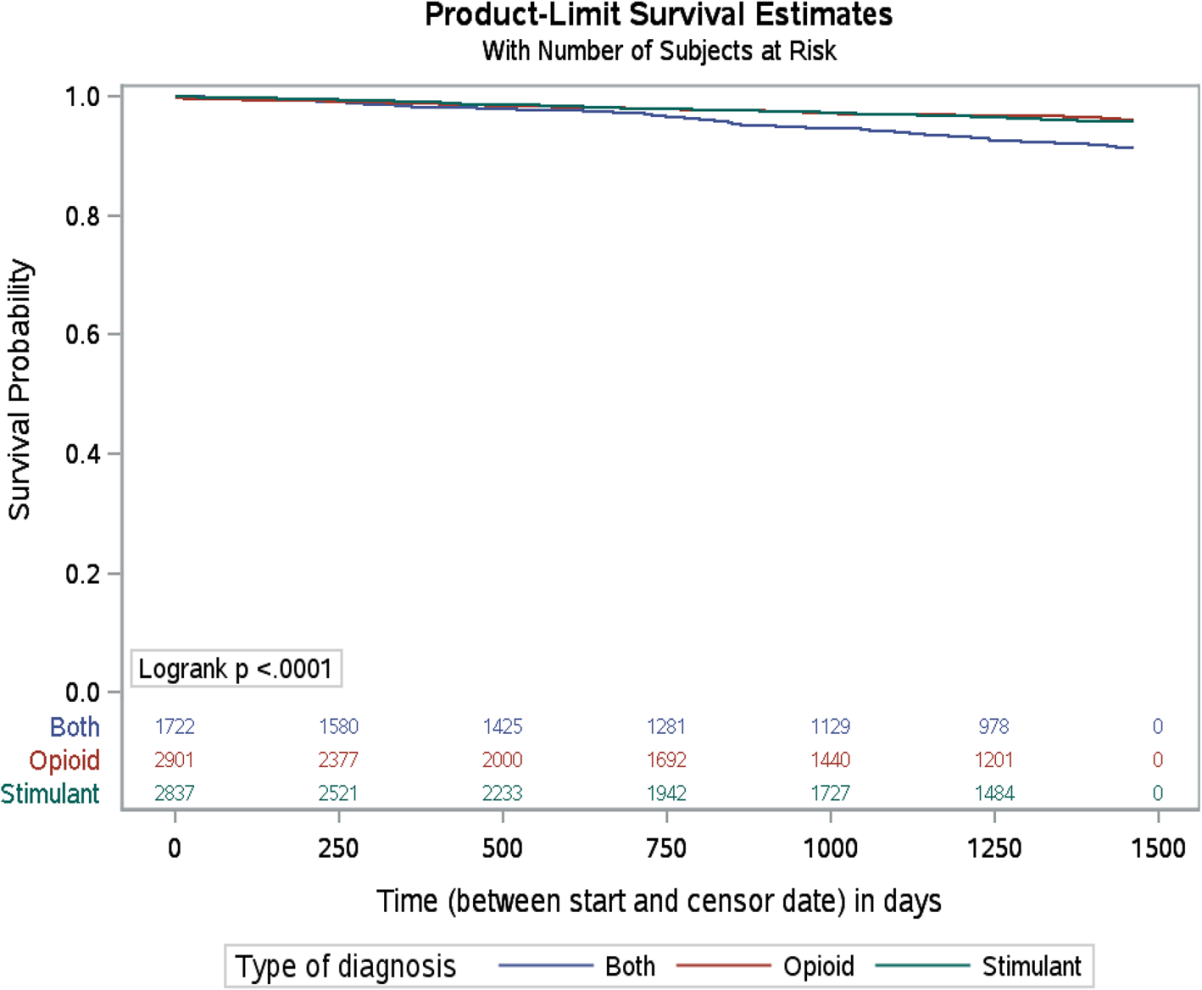
Survival probability by type of diagnosis in BC population **Footnote**: N at risk by SUD Diagnosis type are reported above the X axis. See supplement for further details on N censored and failed by time

The analysis of survival probability revealed a significant difference by substance use type (*p*-value < 0.001). The levels of survival were similar up to two years of follow-up across substance use types, with an increased risk of fatal overdose for participants in the opioid and stimulant use group over time (Fig. 1).

The risk of fatal overdose steadily increased from 2015 to 2018 for people in the opioid and stimulant use group (Table 4). A sensitivity analysis was conducted using a series of cox proportional hazards models with different combinations of covariates and the results remained consistent with those presented in Table 3. OAT is known to have a protective effect on overdose [27] therefore an additional sensitivity analysis was run to estimate the impact of OAT (in the prior 5 years, prior 30 days, and on day of death) on fatal overdose using an adjusted cox model with the same covariates outlined in Table 3. Analyses were repeated by sex and age group. Results in the overall sample were mirrored in females and males. In age stratified analyses, people in the stimulant only group had half the hazard of death compared to those in the opioid only group when considering people aged less than 40. When considering those aged 40 or older, this relationship was reversed, with the stimulant only group having twice the hazard of death compared to the opioid only group. Full model estimates are included in the Supplement.

**Table 4:** Adjusted cox models by time periods.

Substance use type	HR (95%CI)	P-value
**2015-2018 (*N*** **= 7275)**		
Both	2·02 (1·47-2·78)	< 0·0001
Stimulant use	1·05 (0·75-1·48)	0·7644
Opioid use	Reference	
**2016-2018 (*N*** **= 7179)**		
Both	2·16 (1·54-3·02)	< 0·0001
Stimulant use	1·11 (0·77-1·59)	0·5822
Opioid use	Reference	
**2017-2018 (*N*** **= 7042)**		
Both	2·26 (1·52-3·37)	< 0·0001
Stimulant use	0·99 (0·64-1·54)	0·9744
Opioid use	Reference	
**2018 (*N*** **= 6888)**		
Both	2·60 (1·51-4·48)	0·0006
Stimulant use	1·20 (0·66-2·18)	0·5555
Opioid use	Reference	

## Discussion

This study found that people who used both opioids and stimulants had more than twice the hazard of fatal overdose compared to people using opioids only. This finding is consistent with prior studies, whereby the compounded impact of concurrent stimulant and opioid use poses an increased risk of poor outcomes, including discontinuation of OAT [7]. To our knowledge, this is the first Canadian population-level study examining the association between both opioid and stimulant use and illicit drug toxicity (overdose) death. Much of what is known about the use of both opioid and stimulants and overdose is derived from detection of these substance in post-mortem toxicology. In this study, we use a prospective cohort design to identify cases of illicit drug toxicity death following contact with health services for opioid and/or stimulant use.

The descriptive findings suggest that when compared to people who use opioids only, people who use both drugs were significantly less likely to receive all prescribed medication types, including benzodiazepines, z drugs, sedatives, and opioids for pain. This was true despite people who used both opioids and stimulants having significantly more comorbidities. This suggests increased barriers in access to and engagement with health services among this population. Stigma poses a known barrier to service engagement among people who use substances [28] and has been found to be more severe towards people with dual diagnoses [29]. Prior studies have suggested the need for education to reduce provider stigma toward this population and to better understand how to meet service needs [29]. This is particularly important for people who use stimulants, for whom the implementation of harm reduction and treatment interventions remain limited [30].

While much of the framing of overdose risk in North American has focused on opioids, often referred to as the “opioid crisis” [31], our analysis revealed that people who use stimulants have a similar risk of overdose death as compared to people who use opioids. Services remain limited for people who use stimulants in BC, and internationally. Traditionally, interventions available for stimulant use include psychosocial treatments, which have been provided with limited and short-term efficacy [17]. Given the rise of stimulant use and the escalation of overdose deaths in recent years, there has been a call for a new treatment paradigm for stimulant use, paralleling the OUD treatment framework, including prescribed stimulant medications coupled with other health care interventions [30, 32]. In BC and Canada, alternatives to illicit stimulants have been prescribed in a small number of settings and programs, with positive effects. For example, dextroamphetamine has been provided in a clinic in Vancouver since 2016 with observed reductions in illicit stimulant use and improved health outcomes [33, 34]. In 2021, residents of a COVID-19 isolation hotel in Halifax who were using illicit stimulants were provided dextroamphetamine and methylphenidate with no reported cases of overdose or adverse events [35]. Nevertheless, the range of options and reach of this prescribing remains limited, often available only to people with clinical diagnoses of stimulant use disorder and prescribed for daily dispensation at community pharmacies. A wider diversity of alternatives to the illicit drug supply are required to support people with diverse motivations for, patterns of, and goals around their opioid and stimulant use, which may or may not include abstinence [36].

The analysis of survival over time revealed a similar survival probability in the first two years of follow-up across substance use groups, followed by a subsequent decline in survival, the greatest of which was observed in the group using both opioids and stimulants. This finding aligns well with recent data from the US, indicating rising rates of concurrent use among overdose cases over time. For example, between 2012 and 2018, the rate of fatal overdoses that involved cocaine more than tripled and those involving methamphetamine increased almost five-fold [37]. Furthermore, overdose mortality in the US involving both stimulants and opioids has increased more sharply than deaths involving stimulants alone. For example, by 2019, more than 75% of deaths involving cocaine, and approximately half of those involving methamphetamine also involved opioids [38].

The decline in survival over time in BC among people using both opioids and stimulants may reflect a number of missed opportunities for intervention in the time immediately following contact with care for substance use. One such intervention that can be provided for people using opioids and stimulants concurrently is OAT. While widely available in BC, prior studies have demonstrated that concurrent stimulant use is associated with poorer long-term OAT engagement [7]. Our sensitivity analyses revealed that when OAT was added to the model, the hazard of fatal overdose among people who used stimulants and opioids was lower than in the model where OAT was not included. Furthermore, the impact of OAT on overdose mortality did not differ between people using opioids only compared to people using both opioids and stimulants. Studies have shown that where people who use stimulants have been retained in OAT, reductions in stimulant use have been observed over time [8]. This confirms the importance of providing evidence-based effective interventions for OUD to people who use both opioids and stimulants.

Nevertheless, OAT retention rates in BC have been declining in recent years [6]. This decline may reflect that current treatment options are not meeting the preferences of many people who use opioids in BC. For example, more than 70% of people who use opioids and accessed harm reduction sites in BC in 2019 reported smoking opioids [39], and approximately 60% reported heroin as their preferred opioid [36]. These preferences for substances and routes of administration are not met by available treatment options. As such, treatment interventions must be introduced to address the diverse preferences for routes of administration of people who use opioids and stimulants in BC, to include smokable options, such as inhalable diacetylmorphine [40, 41]. Furthermore, it is important to acknowledge that treatment is not always suitable or desired. Scaling up of treatment interventions therefore must be complemented by robust range of harm reduction services. For example, in BC there are several overdose preventions sites and safe consumption services where people can inject, snort, or swallow drugs, however spaces for safe inhalation remain limited [42]. Interventions such as oral OAT will not be sufficient to reduce contacts with the illicit drug supply and reduce overdose risk when they do not meet patients goals and preferences [43]. In this context, efforts must be made to expand OAT to include injectable and inhalable (e.g. hydromorphone and diacetylmorphine) options to meet a wider range of patient preferences [40, 44, 45]. This expansion must be coupled with an accessible and acceptable safe supply [46] of regulated drugs in order to reduce the risk of illicit drug toxicity death resulting from a toxic drug crisis which remains the deadliest public health crisis facing BC and Canada [1, 2].

There are a number of important limitations to this study. First, we report on sex as male or female. This binary variable reflects biological sex assigned to a person at birth, and does not provide information on gender identity. While BC has introduced a third “X” option to its birth certificates, it is not universally accepted and is not yet available in our datasets. Drug types involved in each of the reported deaths are only available for cases deemed as “closed” by the Coroners Service (See supplement). Nevertheless, analysis of illicit drug toxicity deaths in BC has consistently revealed the relevance of both opioids and stimulants to illicit drug toxicity deaths [2]. For example, analyses of all illicit drug toxicity deaths in BC between 2015 and 17 revealed that one or more opioid was found to be relevant to death in 85.5% of cases, and one or more stimulants were found to be relevant to death in 70.6% of cases [47].

In this study, we rely on the use of ICD9/10 codes to identify people with health care visits related to opioid or stimulant use. As such, misclassification of exposure is possible. For example, someone may use opioids and stimulants, but may only have diagnostic codes for one of these substances if both were not recorded during their health care visit. Furthermore, our study does not represent people who may use opioids and or stimulants but who are not in contact with health care. A recent death review panel in BC identified that many people who died of illicit drug toxicity death had not accessed substance use services prior to their death [48]. BC-ODC data show that approximately 51% of deaths between 2010 and 18 had no associated diagnostic codes for substance use disorders. This suggests that the risk of death is present among people who use occasionally or infrequently or who use regularly but do not have a SUD diagnosis. As such, it is possible that the hazard of fatal overdose is underestimated among people who use opioids and stimulants in BC. Nevertheless, given our study is based on a random general population sample, findings may be generalizable to population-level study samples in other North American settings where similar trends in rising opioid and stimulant use have been observed.

## Conclusions

This study has emphasized the elevated risk of fatal overdose facing people who use opioids and stimulants. Expanding access to and increasing support for people who use stimulants is urgently needed in order to reduce risk of overdose mortality in BC. This includes approaches to improving accessibility of treatment such as OAT for people who use stimulants and policy directives to scale up the implementation of evidence-based pharmaceutical alternatives to illicit stimulants. Lessons can be applied more broadly in a North American context where similar trends of opioid and stimulant polysubstance use are being observed.

## Supplementary Information

**Additional file 1.**

## Data Availability

The data used in this study is not publicly available due to privacy considerations. However, researchers can request access to the Provincial Overdose Cohort via annual calls for proposals through Population Data BC (chloe.xavier@bccdc.ca).
