# Supplementary material for: Concurrent use of opioids and stimulants and risk of fatal overdose: A cohort study
Source: BMC Public Health. 2022 Nov 15;22:2084. doi: 10.1186/s12889-022-14506-w (PMC9664696; doi:10.1186/s12889-022-14506-w)
Supplement: Supplementary file 1 — Additional file 1. [file 12889_2022_14506_MOESM1_ESM.docx]

**Supplementary Material**

**Table S1: Exposure: type of substance use disorder diagnosis**

| **Diagnosis** | **ICD9 codes** | **ICD10 codes** | **Algorithm** |
| --- | --- | --- | --- |
| **Substance use disorder** | | | |
| Stimulant use disorder | 3042, 3052, 3044, 3057 | F15, F15 | Any encounter with the health care system with related diagnosis. |
| Opioid use disorder | 3040, 3047, 3055 | F11 | Any encounter with the health care system with related diagnosis. |

Footnote: ICD9 from MSP records (physician billing) and ICD 10 from DAD records (hospitalizations) or NACRS (emergency department visits). See Table S11 for description of full list of data sources.

**Table S2: Outcome measure: Fatal overdose case definition**

| Data Source | Description |
| --- | --- |
| BC Coroners Service | Open investigations (toxicology pending) and closed drug overdose deaths |
| Vital Statistics Deaths | Case defined by ICD version 9 and 10 (e.g., 965.0-poisoning by opiates and related narcotics; E850.0-accidental poisoning by opiates and related narcotics) OR *CARD 9: Cardiac or respiratory arrest/death; CARD 23: Overdose/poisoning (ingestion); CARD 26: Sick; CARD 31: Unconscious |
| Drug-related overdose algorithm | If death date from other administrative databases (e.g., client roster) lies between the start date and end date of an overdose episode, then this overdose episode is defined as a fatal overdose. In these cases, please use the overdose episode start date as the date of the fatal overdose |

**Table S3: Comorbidities: ICD-10 codes used in Elixhauser comorbidity categories**

| **Category** | **ICD-10 codes** |
| --- | --- |
| 1. Congestive heart failure | I099, I110, I130, I193, I255, I420, I425, I426, I427, I428, I429, I43, I50, P290 |
| 1. Cardiac arrhythmia | I441, I442, I443, I456, I459, I47, I48, I49, R000, R001, R008, T821, Z450, Z950 |
| 1. Valvular disease | A520, I05, I06, I07, I08, I091, I098, I34, I35, I36, I37, I38, I39, Q231, Q232, Q233, Z952, Z953, Z954 |
| 1. Pulmonary circulation disorders | I26, I27, I280, I288, I289 |
| 1. Peripheral vascular disorders | I70, I71, I731, I738, I739, I771, I790, I792, K551, K558, K559, Z958, Z959 |
| 1. Hypertension uncomplicated | I10 |
| 1. Hypertension complicated | I11, I12, I13, I15 |
| 1. Paralysis | G041, G114, G801, G802, G81, G82, G830, G831, G832, G833, G834, G839 |
| 1. Other neurological disorders | G10, G11, G12, G13, G20, G21, G22, G254, G255, G312, G318, G319, G32, G35, G36, G37, G40, G41, G931, G934, R470, R56 |
| 1. Chronic pulmonary disease | I278, I279, J40, J41, J43, J44, J45, J46, J47, J60, J61, J62, J63, J64, J65, J66, J67, J684, J701, J703 |
| 1. Diabetes uncomplicated | E100, E101, E109, E110, E111, E119, E120, E121, E129, E130, E131, E139, E140, E141, E149 |
| 1. Diabetes complicated | E10-E0E108, E112-E118, E122-E128, E132-E138, E14-E148 |
| 1. Hypothyroidism | E00-E03, E890 |
| 1. Renal Failure | I120, I131, N18, N19, N250, Z490, Z491, Z492, Z940, Z992 |
| 1. Liver disease | B18, I85, I865, I982, K70, K711, K713, K714, K715, K717, K72, K73, K74, K760, K762-K769, Z94 |
| 1. Peptic ulcer excluding bleeding | K257, K259, K267, K269, K277, K279, K287, K289 |
| 1. HIV/AIDS | B20, B21, B22, B24 |
| 1. Lymphoma | C81-C85, C88, C96, C900, C902 |
| 1. Metastic cancer | C77-C80 |
| 1. Solid tumor without metastasis | C00-C26, C30-C34, C37-C41, C43, C45-C58, C60-C76, C97 |
| 1. Rheumatoid arthritis/collagen | L940, L941, L943, M05, M06, M08, M120, M123, M30, M310, M311, M312, M313, M32, M33, M34, M35, M45, M461, M468, M469 |
| 1. Coagulopathy | D65, D66, D67, D68, D691, D693, D694, D695, D696 |
| 1. Obesity | E66 |
| 1. Weight loss | E40-E46, R634, R64 |
| 1. Fluid and electrolyte disorders | E222, E86, E87 |
| 1. Blood loss anemia | D500 |
| 1. Deficiency anemia | D508, D509, D51, D52, D53 |
| 1. Drug abuse | F11.x–F16.x, F18.x, F19.x,  Z71.5, Z72.2 |
| 1. Psychoses | F20.x, F22.x–F25.x, F28.x,  F29.x, F30.2, F31.2, F31.5 |
| 1. Depression | F20.4, F31.3–F31.5, F32.x,  F33.x, F34.1, F41.2, F43.2 |

Footnote: Based on ICD-10 codes categorised by Quan et al^[[1]](#footnote-1)^

**Table S4: Sensitivity analyses of various covariate combinations on the outcome of fatal overdose**

| **Model** | **Variables included** | **HR (95% CI)**  **Opioid use disorder only vs both** | **HR (95% CI)**  **Stimulant use disorder only vs both** | **AIC** |
| --- | --- | --- | --- | --- |
| 1 | Type of diagnosis | 0.48(0.36-0.65) | 0.50(0.38-0.67) | 4621.3 |
| 2 | Model 1 + Sex, age | 0.48 (0.35-0.64) | 0.49(0.37-0.65) | 4598.8 |
| 3 | Model 2 + Comorbidity Index | 0.47(0.35-0.64) | 0.49(0.37-0.65) | 4610.7 |
| 4 | Model 3 + mental characteristics (bipolar, schizophrenia, personality disorder, stress disorder) | 0.48(0.35-0.65) | 0.48(0.36-0.63) | 4615.2 |
| 5 | Model 4 + mental characteristics (depression, anxiety) | 0.48(0.35-0.65) | 0.47(0.36-0.63) | 4616.3 |
| 6 | Model 5 + Injection drug use | 0.49(0.36-0.66) | 0.49(0.37-0.65) | 4616.3 |
| 7 | Model 6 + OAT | 0.49(0.36-0.67) | 0.49(0.36-0.66) | 4618.3 |
| 8 | Model 7 + benzo, z drug, opioids for pain | 0.48(0.35-0.65) | 0.50(0.36-0.67) | 4619.8 |
| 9 | Model 8 + sedatives | 0.48(0.35-0.65) | 0.50(0.37-0.68) | 4622.9 |
| 10 | Model 9 + chronic pain | 0.48(0.35-0.66) | 0.50(0.37-0.67) | 4624.4 |

**Footnote**: HR= hazard ratio; CI= confidence interval; AIC= Akaike Information Criterion; OAT= opioid agonist treatment

**Table S5: Sensitivity analysis excluding stimulant only group, and testing effect of OAT on the outcome**

|  | **HR (95% CI)** | **P value** |
| --- | --- | --- |
| **Type of diagnosis** |  |  |
| Both | 1.87(1.34-2.58) | 0.0002 |
| Opioid | Reference |  |
| **Sex-Female** | 0.52(0.38-0.71) | <0.001 |
| **Age** |  |  |
| <30 | 1.21(0.79-1.85) | 0.3847 |
| 30-39 | 0.84(0.55-1.29) | 0.430 |
| 40-49 | 1.01(0.67-1.53) | 0.952 |
| 50+ | Reference |  |
| **Health Authority** |  |  |
| Fraser | 0.68(0.47-0.99) | 0.046 |
| Interior | 0.91(0.59-1.40) | 0.6556 |
| Northern | 0.80(0.40-1.61) | 0.5290 |
| Vancouver Island | 088(0.55-1.40) | 0.5896 |
| Vancouver Costal | Reference |  |
| **Comorbidity Index** |  |  |
| None | 0.98(0.66-1.45) | 0.912 |
| 1 | 0.77(0.50-1.20) | 0.2414 |
| 2 | 0.66(0.42-1.04) | 0.0756 |
| 3+ | Reference |  |
| **Benzodiazepine** | 1·18(0·79-1·78) | 0.4153 |
| **Z-drugs** | 1.25(0.74-2.11) | 0.4049 |
| **Sedatives** | 0.89(0.57-1.38) | 0.6043 |
| **Opioids for pain** | 0.94(0.62-1.46) | 0.8107 |
| **OAT** | 0.85(0.56-1.27) | 0.4136 |

**Footnote**: HR= hazard ratio; CI= confidence interval; AIC= Akaike Information Criterion; OAT= opioid agonist treatment

**Table S6: Sensitivity analysis excluding stimulant only group, and testing effect of OAT on the outcome (OAT defined three ways)**

|  | **Original model**  **(Table S5)**  **HR (95% CI)** | **Sensitivity**  **Model 1**  **HR (95% CI)** | **Sensitivity**  **Model 2**  **HR (95% CI)** | **Sensitivity**  **Model 3**  **HR (95% CI)** |
| --- | --- | --- | --- | --- |
| **Type of diagnosis** |  |  |  |  |
| Both | **1.87(1.34-2.58)** | **1.89(1.36-2.62)** | **2.01(1.44-2.78)** | **2.04(1.47-2.83)** |
| Opioid | Reference | Reference | Reference | **Reference** |
| **OAT** |  |  |  |  |
| Prior 30 days at baseline | 0.85(0.56-1.27) | - | - | **-** |
| Prior 5 years at baseline | - | 1.28(0.92-1.78) | - | **1.51(1.10-2.08)** |
| On day of death | - | - | **0.14(0.07-0.30)** | **0.14(0.07-0.28)** |

**Footnote**: HR= hazard ratio; CI= confidence interval; AIC= Akaike Information Criterion; OAT= opioid agonist treatment

Models are all adjusted for age, sex, health authority, comorbidity index, benzo, z drugs, sedatives, opioids for pain).

**Table S7: Association of SUD type on the outcome of fatal overdose, by sex**

|  | **Unadjusted Estimates** | | **Adjusted Estimate** | |
| --- | --- | --- | --- | --- |
|  | HR (95%CI) | P-value | HR (95%CI) | P-value |
| **Female (N=2852)** |  |  |  |  |
| Substance use type | | | | |
| Both | 2·21 (1·27-3·83) | 0·005 | 2·13 (1·15-3·95) | 0·016 |
| Stimulant | 1·10 (0·61-2·00) | 0·7545 | 1·17(0·60-2·29) | 0·6434 |
| Opioid | Reference | | | |

|  | **Unadjusted Estimates** | | **Adjusted Estimate** | |
| --- | --- | --- | --- | --- |
|  | HR (95%CI) | P-value | HR (95%CI) | P-value |
| **Male (N=4423)** |  |  |  |  |
| Substance use type | | | | |
| Both | 1·77 (1·25-2·51) | 0·001 | 2·13 (1·15-3·95) | 0·016 |
| Stimulant | 0·88 (0·61-1·26) | 0·4799 | 1·17(0·60-2·29) | 0·6434 |
| Opioid | Reference | | | |

**Table S8: Association of SUD type on the outcome of fatal overdose, by age**

|  | **Unadjusted Estimates** | | **Adjusted Estimate** | |
| --- | --- | --- | --- | --- |
|  | **HR (95%CI)** | **P-value** | **HR (95%CI)** | **P-value** |
| **<40 years (N=3817)** |  |  |  |  |
| Substance use type | | | | |
| Both | 1·43 (0·94-2·18) | 0·0937 | 1·27 (0·81-2·00) | 0·2919 |
| Stimulant | 0·60 (0·38-0·96) | 0·0316 | 0·47(0·28-0·78) | 0·0033 |
| Opioid | Reference | | | |

|  | **Unadjusted Estimates** | | **Adjusted Estimate** | |
| --- | --- | --- | --- | --- |
|  | **HR (95%CI)** | **P-value** | **HR (95%CI)** | **P-value** |
| **≥40 (N=3458)** |  |  |  |  |
| Substance use type | | | | |
| Both | 2·64 (1·75-4·00) | < 0·001 | 2·83 (1·82-4·41) | <0.001 |
| Stimulant | 1·56 (1·03-2·38) | 0·0382 | 1·98(1·26-3·12) | 0·003 |
| Opioid | Reference | | | |

**Table S9: Comparison of characteristics of participants included and excluded in final model**

|  | **Included**  **N(%)** | **Excluded**  **N(%)** | **Total**  **N(%)** | **P value** |
| --- | --- | --- | --- | --- |
|  | 7275(97·5) | 185(2·5) | 7460(100·0) |  |
| **Type of use** |  |  |  |  |
| Both | 1712(23·5) | 10(5·4) | 1722(23·1) | <0.001 |
| Stimulant | 2759(38·0) | 142(76·8) | 2901(38·9) |  |
| Opioid | 2804(38·5) | 33(17·8) | 2837(38·0) |  |
| **Sex** |  |  |  |  |
| Female | 2825(38·8) | 48(26·0) | 2873(38·5) | <0.001 |
| **Age** |  |  |  |  |
| <30 | 1883(25·9) | 53(28·7) | 1936(26·0) | <0.001 |
| 30-39 | 1934(26·6) | 76(41·1) | 2010(26·9) |  |
| 40-49 | 1548(21·3) | 40(21·6) | 1588(21·3) |  |
| 50+ | 1910(26·2) | 16(8·7) | 1926(25·8) |  |
| **Comorbidities** |  |  |  |  |
| **Elixhauser index** |  |  |  |  |
| 0 | 3006(41·3) | 159(86·0) | 3165(42·4) | <0.001 |
| 1 | 1405(19·3) | 7(3·8) | 1412(18·9) |  |
| 2 | 1284(17·7) | 9(4·9) | 1293(17·3) |  |
| 3+ | 1580(21·7) | 10(5·4) | 1590(21·3) |  |
| **Prescribed medications (Prior 30 days at baseline)** |  |  |  |  |
| Benzodiazepines | x | x | 995(13·3) | <0.001 |
| Z drugs | x | x | 458(6·1) | 0.001 |
| Sedatives | x | x | 1322(17·7) | <0.001 |
| Opioid for pain | 4244(58·3) | 21(11·4) | 4265(57·2) | <0.001 |

Footnote: Participants who were excluded were N=185 due to missing on the health authority variable. The "X" reflects data that are suppressed due to small cell sizes <5.

**Table S10: Drugs deemed “relevant to death” according to BC Coroners Service toxicology data**

| **Drug detected** | **Both**  **N=52**  **N(%)** | **Opioid**  **N=31**  **N(%)** | **Stimulant**  **N=49**  **N(%)** |
| --- | --- | --- | --- |
| Opioids |  | 10(32·3) | x |
| Opioid and other | x | x | x |
| Opioid and stimulant | 35(67·3) | 12(38·7) | 23(46·9) |
| opioid and stimulant and other | 10(19·2) | 7(22·6) | 8(16·3) |
| Stimulants | x | x | 8(16·3) |
| Stimulants and other | x | x | x |
| **Total** | **%** | **%** | **%** |
| % Deaths with opioids detected | 98·1% | 96·8% | 75·5% |
| % deaths with stimulants detected | 88·5% | 64·5% | 87·8% |
| % deaths with opioids and stimulants detected | 86·5% | 61·3% | 63·3% |

**Footnote**: Toxicology data are only available for cases of death listed as “closed” by the BC Coroners Service (N=132), of all the deaths observed in the study, data were available for N=52 of the deaths in the both group, N=31 in the opioid only group, and N=49 in the stimulant only group. Other drugs are those not classified as stimulants or opioids, and include benzodiazepines, alcohol, and cannabis. The "X" reflects data that are suppressed due to small cell sizes <5.

**Table S11: Prescribed medications**

| **Benzodiazepines** |  |
| --- | --- |
|  | alprazolam |
|  | bromazepam |
|  | chlordiazepoxide |
|  | clobazam |
|  | clonazepam |
|  | clorazepate |
|  | diazepam |
|  | flurazepam |
|  | lorazepam |
|  | nitrazepam |
|  | oxazepam |
|  | temazepam |
|  | triazolam |
| **Z drugs** | zaleplon |
|  | zolpidem |
|  | zopiclone |
| **Sedatives** | Alprazolam |
|  | amitriptyline |
|  | asenapine |
|  | Baclofen |
|  | Bromazepam |
|  | Buprenorphine |
|  | Butorphanol |
|  | Carbamazepine |
|  | Chloral |
|  | Chloradiazepoxide |
|  | Clomipramine |
|  | clonazepam |
|  | Clozapine |
|  | Codeine |
|  | Cyclobenzaprine |
|  | desipramine |
|  | Diazepam |
|  | doxepin |
|  | Fentanyl |
|  | Flupentixol |
|  | Gabapentin |
|  | Hydromorphone |
|  | Imipramine |
|  | Lamotrigine |
|  | Levetiracetam |
|  | Lorazepam |
|  | Loxapine |
|  | maprotiline |
|  | Meperidine |
|  | Methadone |
|  | methocarbamol |
|  | Methotrimeprazine |
|  | mirtazapine |
|  | morphine |
|  | Nabilone |
|  | nefazodone |
|  | Nitrazepam |
|  | nortriptyline |
|  | Olanzapine |
|  | Oxazepam |
|  | Oxycodone |
|  | Pentazocine |
|  | Periciazine |
|  | phenelzine |
|  | Phenobarbital |
|  | Phenytoin |
|  | Pipotiazine |
|  | Pregabalin |
|  | Prochlorperazine |
|  | Propoxyphene |
|  | Quetiapine |
|  | Respiridone |
|  | Tapentadol |
|  | Temazepam |
|  | Thioridazine |
|  | Topiramate |
|  | Tramadol |
|  | tranylcypromine |
|  | trazodone |
|  | Triazolam |
|  | trimipramine |
|  | tryptophan |
|  | Valproic acid |
|  | Ziprasidone |
|  | Zolpidem |
|  | Zoplicone |
|  | Zuclopenthixol |
| **Opioids for pain** |  |
|  | buprenorphine |
|  | butorphanol |
|  | codeine |
|  | fentanyl |
|  | hydromorphone |
|  | meperidine |
|  | methadone |
|  | morphine |
|  | oxycodone |
|  | pentazocine |
|  | propoxyphene |
|  | tapentadol |
|  | tramadol |
| ***OAT*** | Methadone |
|  | Buprenorphine |
|  | Buprenorphine/naloxone |
|  | Slow-release oral morphine |

**Figure S1: Overtime fatal overdose by type of diagnosis**

**Footnote**: Figure S1 shows that number of people died from overdose steady increase between 2015 and 2018. Except 2016, the majority of deaths were attributed to the group with concurrent disorder with larger proportion in years 2017 and 2018. However, the difference was not significant (p-value 0.71).

**Figure S2: Participants censored by time in the Kaplan Meier Survival analysis, by SUD diagnosis type**

**Figure S3: Participants failed by time in the Kaplan Meier Survival analysis, by SUD diagnosis type**

**Table S12: Participants failed and censored by time in the Kaplan Meier Survival analysis, by SUD diagnosis type**

| **Time** | **Both** | | **Opioid** | | **Stimulants** | |
| --- | --- | --- | --- | --- | --- | --- |
|  | **Censored** | **Failed** | **Censored** | **Failed** | **Censored** | **Failed** |
| 0 | 0 | 0 | 0 | 0 | 0 | 0 |
| 250 | 142 | 17 | 524 | 25 | 316 | 17 |
| 500 | 155 | 34 | 377 | 39 | 288 | 34 |
| 750 | 144 | 52 | 308 | 49 | 291 | 53 |
| 1000 | 152 | 77 | 252 | 62 | 215 | 64 |
| 1250 | 151 | 100 | 239 | 67 | 243 | 73 |
| 1500 | 0 | 111 | 0 | 74 | 0 | 87 |

**Footnote**: Failed= those who had the event. Censored = those left in the study at each time point. SAS rounded the last instance of time to 1500, last failed participant was on the following days: Both, 1436; Opioid; 1441; Stimulant; 1437.

**Table S13:** **Data Sources and descriptions**

| **Data source** | **Description** |
| --- | --- |
| **BC Coroner’s Service (BCCS)** | The BCCS investigates all accidental and undetermined illicit drug-related overdose deaths in BC. Data contains information about date of death/injury, age group, sex of decedent, location of death, location of residence of decedent, drug use and route of administration, and toxicology results. |
| **BC Emergency Health Services (BCEHS)** | The BCEHS data contains information about the time and location of an overdose event, demographic information about the patient, and details from the dispatch and paramedic’s assessment, treatment, and transportation of patients, including whether or not naloxone was administered by paramedics. The data uses impression codes from the BCEHS Patient Care Report (PCR) data to identify overdose-related events. The BCEHS data includes various coding combinations used by first responders in the PCR data. The PCR originated from paper-based reporting completed by paramedics on-scene and entered into the Patient Care Information System (PCIS). As of April 2019, virtually all paramedic records in BC will be entered in electronic forms into the new SIREN system, which has additional impression codes for overdose-related events. The 2019 Cohort includes information from both PCIS and SIREN. |
| **Drug and Poison Information Centre (DPIC)** | Contains information about calls to the DPIC from the public or medical personnel for clinical advice on poisoning management. The DPIC data include date and time of call, age and sex of patient, postal code and city of call origin, drugs associated with the call, route of drug administration, symptoms, and outcome. |
| **National Ambulatory Care Reporting System (NACRS)** | The NACRS at the Canadian Institute for Health Information (CIHI) is a national database designed to capture information on patient visits to hospital-based and community-based ambulatory care. Data are collected at the time of service from participating facilities and includes the following data elements: demographic, clinical, administrative, financial, and service-specific information including information on discharges, deaths, and transfers from facilities within a fiscal year (April 1 to March 31). |
| **Medical Services Plan (MSP)** | Contains records of all fee-for-service provider visits billed to the province’s universal health insurance program. This excludes services by providers on alternative payment plans. Providers are classified as physicians or supplementary benefit practitioners (e.g., physiotherapists). Information provided about a visit includes patient demographics, service date, service location, provider type, and reason for the visit using IDC-9 coding schemes. |
| **Discharge Abstract Database (DAD)** | The DAD data captures discharges, transfers, and deaths occurring in acute care hospitals in BC. Information about a patient stay includes patient demographics, timing of the stay, location of service, provider type, and information on health conditions and procedures relevant to the stay. Diagnostic coding uses ICD-10 codes. |
| **Vital Statistics Deaths (VSD)** | VSD captures cause of death information from residents of BC who have died in BC, and includes demographic and diagnostic information, whether the Coroner was notified and examined the body, and date and location of death. Diagnostic coding uses ICD-10 codes. |
| **PharmaNet (PNET)** | Contains records of all ambulatory care prescription dispensations in the province of BC. Information about a dispensation includes patient demographics, geographic location of dispensation, type of prescriber, drug specific information, formulation, and dose. |
| **Client Roster (CLR)** | Contains demographic characteristics, death date, and location information (including latitude and longitude, and postal code) of BC residents for each calendar year available after 2010. |

1. Quan, H., Sundararajan, V., Halfon, P., Fong, A., Burnand, B., Luthi, J. C., ... & Ghali, W. A. (2005). Coding algorithms for defining comorbidities in ICD-9-CM and ICD-10 administrative data. *Medical care*, 1130-1139. [↑](#footnote-ref-1)
